# Boswellic Acids as Effective Antibacterial Antibiofilm Agents

**DOI:** 10.3390/molecules27123795

**Published:** 2022-06-13

**Authors:** Petr Jaroš, Elizaveta Timkina, Jana Michailidu, Dominik Maršík, Markéta Kulišová, Irena Kolouchová, Kateřina Demnerová

**Affiliations:** 1 Department of Biochemistry and Microbiology, University of Chemistry and Technology, 16628 Prague, Czech Republic; jarose@vscht.cz (P.J.); demnerok@vscht.cz (K.D.); 2 Department of Biotechnology, University of Chemistry and Technology, 16628 Prague, Czech Republic; michailj@vscht.cz (J.M.); marsikd@vscht.cz (D.M.); kulisovm@vscht.cz (M.K.); hanzliki@vscht.cz (I.K.)

**Keywords:** boswellic acid, microbial biofilm, bacterial biofilm, antibacterial, FIC, antibiotic

## Abstract

Boswellic acids are biologically active pentacyclic terpenoid compounds derived from *Boswellia* sp. plants. Extracts containing these acids have a number of positive effects on human health, especially in the treatment of inflammation, arthritis, or asthma. With increasing resistance to common antibiotics, boswellic acid-containing extracts could serve as an alternative or work in synergy with commonly available preparations. This study aims to determine the effect of boswellic acids on suspension cells and biofilms of *Staphylococcus epidermidis*, *Enterococcus faecalis*, and *Escherichia coli.* The antimicrobial and antibiofilm effect found was compared with commonly available antibiotics to control these undesirable microorganisms. The synergistic effect of boswellic acids and common antibiotics on the growth of these microorganisms was also determined. All tested microorganisms showed a positive additive effect of antibiotics and boswellic acid extract. The most significant effect was found in *Enterococcus faecalis* ATCC 29212 in a combination of 0.2 × MIC80 erythromycin (0.2 mg/L) and 0.8 × MIC80 boswellic acid extract (16 mg/L).

## 1. Introduction

Many resistant bacteria are preferably found in the form of a biofilm, which is between 10 and 1000 times more resistant to antimicrobials than planktonic cells [1] and can colonize a variety of surfaces and persist in a variety of environments—agriculture [2], the food industry [3], biocorrosion and shipbuilding [4,5], in medicine catheters [6], orthopedic prosthetics [7], or cardiovascular equipment [8]. Modern medicine faces the issue of increasing antibacterial resistance reducing the effectiveness of antibiotics due to the growing spread of resistant bacteria. It is estimated that 0.7 million people die each year from diseases caused by resistant strains [9]. The body responds to microbial infection or inflammation in various ways, such as fatigue, frequent inflammation, or gastrointestinal problems, that are commonly treated by steroid or non-steroidal drugs which can lead to side effects with long-term use [10].

The development of the resistance of bacterial strains necessitates the use of new antimicrobials or drugs. The ideal antimicrobial agent should selectively reduce the virulence factors of the microorganism without adversely affecting the natural microflora and the tissue cells themselves. Promising antimicrobial agents include natural substances [11]. Five classes of natural compounds with antimicrobial and antibiofilm properties have been reported—lectins, alkaloids, polypeptides and polyacetylenes, terpenoids, phenols, and essential oils [12,13]. The active components of essential oils include terpenes, with a pentacarbon isoprene unit [14]. Terpenes are metabolized by the mevalonate pathway and control the expression of inflammatory genes [15].

Among the so far scarcely studied plants with antimicrobial and antibiofilm abilities used for millennia is the genus *Boswellia*, belonging to the *Burseraceae* family, with 43 different species of *Boswellia* described, which differ in the effectiveness of essential oils (e.g., *B. carteri, B. papyrifera*, *B. serrata,* and *B. rivae*) [16]. *B. serrata* is one of the most widely used in both the food and food perfume and flavor industries [17]. Boswellia’s active ingredients include boswellic acids (triterpenes and cembranoids-diterpenes). Boswellic acids account for between 25 and 35% of the resin content. Boswellic acid, 11-keto-β-boswellic acid, and acetyl-11-keto-β-boswellic acid are involved in the apoptosis of cancer cells (brain tumors, leukemic cells, colon cancer cells) [18]. All of these substances are characterized by a high biological activity [19]. *Boswellia* ethanol extract is used to treat arthritis [20,21], inflammatory diseases [22,23], and asthma [24]. *Boswellia* extract is effective against Gram-negative bacteria, which cause aggressive periodontitis [25]. The addition of *B. serrata* extract is also effective in the local treatment of burns with *Staphylococcus aureus* MRSA infection [26].

Among the important producers of biofilm from Gram-positive bacteria are mainly members of the *Staphylococcaceae* family with the most well-known pathogenic species *S. aureus* in the foreground. Another important family includes representatives of *Enterococcaceae*, whose research focuses mainly on pathogenic strains of *E. faecium* and *E. faecalis*. Although these species are often native commensals of humans, biofilm formation can be a key factor in the formation of virulent strains and can, thus, lead to the emergence of multidrug resistance. *Staphylococssus* spp. is an aerobic to facultatively anaerobic microorganism that produces a variety of envelope polysaccharides which give rise to a capsule that renders these bacteria resistant to opsonization and phagocytosis in the human body [27]. In the case of *S. aureus* and *S. epidermidis*, polysaccharide layer formation has been found to significantly assist cells in the early stages of biofilm formation [28]. *S. aureus* and *S. epidermidis* are the most pathogenic species of this genus. The genus *Enterococcus* belongs to the family *Enterococcaceae*. These are non-sporulating, facultatively anaerobic cocci that occur either singly, in pairs, or in chains [29]. In humans, they colonize mainly the oral cavity, female genitalia, and gastrointestinal tract. Both *E. faecalis* and *E. faecium* are the most abundant enterococci in the intestinal microflora. *E. faecalis* is responsible for up to 80% of human enterococcal infections [30,31]. They are the third most common nosocomial pathogens causing systemic bacteraemias, peritonitis, endocarditis, urinary tract infections, open wound infections, and infections caused by the colonization of implants and catheters, similar to *S. epidermidis* [31,32,33].

Gram-negative bacteria are more resistant to antibiotics (e.g., vancomycin) than Gram-positive cells due to the relative impermeability of the outer membrane [34]. Homoserine lactones are a signal molecule of quorum sensing (QS) in Gram-negative bacteria. As the bacterial density increases, the secretion of the signaling molecule increases, and upon reaching a certain concentration, the signaling molecule binds to its receptor and activates the synthesis of extracellular polysaccharides and toxic substances [35]. Important representatives of Gram-negative bacteria include *E. coli*, mostly commensal and non-pathogenic, facultatively anaerobic, immobile or motile bacteria. *E. coli* is an important element of the normal intestinal microflora of animals and humans. However, some strains are pathogenic and are classified into several pathotypes. These pathotypes are characterized by a number of virulence factors, toxin production, epithelial adhesion, and surface colonization. Both pathogenic and commensal *E. coli* form biofilms in the gastrointestinal tract and are controlled by the extracellular matrix and adhesion molecules [36,37].

The aim of this study was to evaluate the antimicrobial (antibiofilm) activity of boswellic acids against the Gram-positive bacteria *Staphylococcus epidermidis* and *Enterococcus faecalis* and the Gram-negative bacteria *E. coli*. The antimicrobial and antibiofilm effects of boswellic acids have been compared with conventional antibiotics for the treatment of infections caused by these pathogens.

## 2. Results and Discussion

Since the discovery of penicillin, antibacterial drugs have become an integral part of the treatment of infections [38]. Due to the use of a large number of antibiotics and various biocides in hospitals, highly resistant strains of *E. faecalis*, *E. faecium*, and *S. epidermidis* have recently started to appear [31]. These are the third most common nosocomial pathogens causing systemic bacteraemias, peritonitis, endocarditis, urinary tract infections, open wound infections, and infections due to implant and catheter colonization [31,32,33]. This phenomenon is determined by naturally occurring genes for resistance to a number of antibiotic types, along with resistance acquired by horizontal genetic exchange [39]. Acquired resistance in multidrug-resistant strains includes resistance to high concentrations of β-lactams and aminoglycosides, glycopeptides (vancomycin, teicoplanin), tetracyclines, macrolides, chloramphenicol, and other antibiotics [29,30]. Resistance to glycopeptides is a significant problem, as they are the so-called antibiotics of last resort. These are substances that are used only in cases where treatment with other antibiotics has failed due to their frequent side effects [33]. Due to the increasing resistance to glycopeptides, these substances are gradually being replaced by linezolid and daptomycin [40].

Compared to synthetic drugs, herbal products are often safer to use [41,42]. As much as 80% of the population living in the developing world is reported to use plant products for the treatment of various types of diseases [43]. The mechanisms of action of natural substances on the biofilm include membrane disruption, the ability to bind to the adhesin-cell wall complex, interactions with DNA, and protein binding [44,45].

A number of natural substances are currently being studied for their antimicrobial effects [11]. Not only are the natural substances from the plants themselves studied but also the extract of agents for the antimicrobial effect of such preparations [44] or the effect of plant origin on the strength of the antimicrobial effect [46]. The monitored plants include B. serrata, for which, in addition to the method of extraction of antimicrobial substances, the strength of the antimicrobial effect may also depend on the origin of the plant. B. serrata has been newly tested, for example, against malaria parasites [47], as an antimicrobial adjuvant in packaging films [48], and also as a feed additive with a positive effect on the intestinal microflora of broilers, rabbits, and fish [49,50,51,52].

In this work, the effect of B. serrata extract containing 35% boswellic acids on *S. epidermidis*, *E. faecalis,* and *E. coli* was studied. The antimicrobial effect of the extract was compared with that of erythromycin (ERM) for Gram-positive cocci and polymyxin B (PMB) for Gram-negative bacteria. We compared the effect of substances on bacterial agents for both suspension and biofilm growth (Table 1).

The MIC50 values of bacterial suspension cells were low, in the range between 0.3 mg/L and 0.5 mg/L for the antibiotics ERM and PMB. ERM is a macrolide antibiotic that inhibits protein synthesis and is used to treat many bacterial diseases, such as listeriosis, diphtheria, atypical pneumonia (mycoplasma, chlamydia), and some intestinal diseases [53]. PMB is a polypeptide antibiotic that disrupts cell membrane permeability due to its interaction with its phospholipids and is also one of the potential solutions for bacterial biofilm destruction [54]. All examined bacterial strains were significantly more sensitive to the effects of antibiotics during suspension growth than the natural Boswellia extract (10–100 mg/L). According to previous studies, boswellic acids have been found to act as non-competitive 5-lipoxygenase inhibitors [55]. A similar trend as for MIC50 values was observed for MIC80 values, where the inhibitory concentration of ERM was in the range from 0.5–1 mg/L for both antibiotics and from 20–150 mg/L for BOSW depending on the bacterial species when *E. coli* strains were more resistant to the effects of natural extract. In an experimental study describing the effect of pure boswellic acids on the same microorganisms, an MIC80 of up to 128 mg/L for acetyl-β-boswellic acid (ABA) was not determined for E. faecalis, but for 3-acetyl-11-keto-β-boswellic acid (AKBA) MIC80 was 4 mg/L [18]. Given that BOSW contains at least 35% of both of these acids in combination, the extract we tested could be considered more effective. Moreover, a study by Weckesser [56] confirms the antimicrobial effects of *B. serrata* resin extract against the studied suspension populations of Streptococcus and *Escherichia coli*. The different levels of the antimicrobial effect of vegetable essential oils against different bacterial strains have also been described [57]. A significantly weaker antimicrobial effect on Staphylococcus, Enterobacter, and Streptococcus was found with boswellic acid extract from *B. dalzielii* (3 mg/mL) [58], confirming the findings of Schillaci [59], which states that the antimicrobial effect of extracts from *Boswellia* sp. varies depending on the species of this plant.

**Figure 1:**
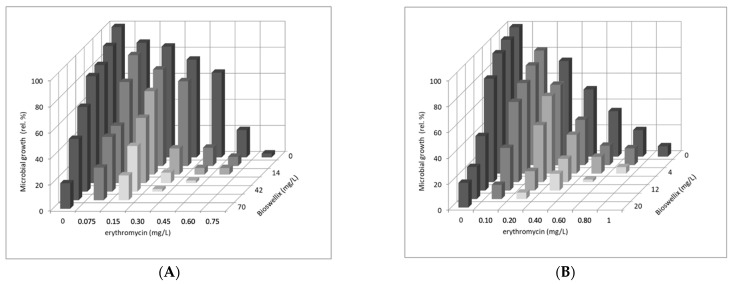

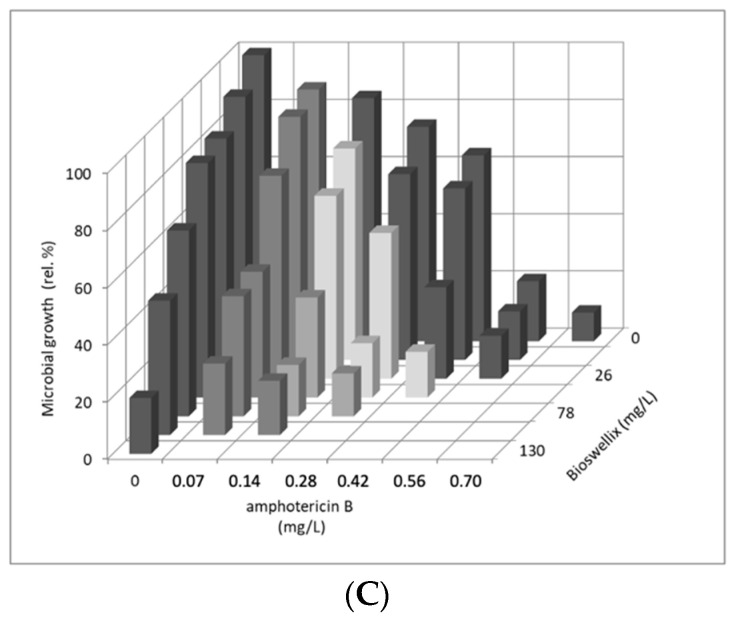
Effect of erythromycin or polymyxin B in combination with boswellic acids extract on suspension growth of (**A**) *S. epidermidis* ATCC 14990, (**B**) *E. faecalis* ATCC 29212, and (**C**) *E. coli* CCM 3954. *x*- and *z*-axes depict fractions of MIC80 of respective compound. *y*-axis depicts microbial growth of suspension cells in relative percentages (control represents 100%).

Due to the interesting results of *B. serrata* extract on selected bacterial representatives, the combined effect of antibiotic and BOSW on suspension cells of *S. epidermidis* ATCC 14990, E. faecalis ATCC 29212, and *E. coli* CCM 3954 was further studied. The results are shown in Figure 1A–C.

Although the antimicrobial effects of *B. serrata* extracts are well known, their effects in combination with antibiotics have not yet been studied. The interaction of both erythromycin and polymyxin B with boswellic acid extract had an additive effect on the inhibition of suspension growth of all tested bacterial strains (Figure 1). The strongest additive effect was observed in *E. faecalis* ATCC 29212 in a combination of 0.2 × MIC80 erythromycin (0.2 mg/L) and 0.8 × MIC80 boswellic acid extract (16 mg/L). It can be seen that boswellic acid extract significantly reduced the effective antibiotic concentration compared to MIC80. There was also an effective reduction in the effective antibiotic concentration in *S. epidermidis* ATCC 14990 (Figure 1A) with a combination of 0.4 × MIC80 erythromycin (0.3 mg/L) and 0.6 × MIC80 boswellic acid extract (42 mg/L). The combination of polymyxin B and boswellic acid extract was the least effective on Gram-negative *E. coli* CCM 3954, where the most effective combinations were 0.2 × MIC80 polymyxin B (0.14 mg/L) and 0.6 × MIC80 boswellic acid extract, where the growth decreased to 18 rel%. Samreen [60] performed in silico screening of 19 phytochemicals against the AcrB efflux pump of *E. coli*, responsible for the multi-resistant efflux pump (MDR) in Gram-negative bacteria. They evaluated chlorogenic acid as the most effective substance, which was also the most effective in a combination therapy with tetracycline. The use of a complex extract may be more advantageous due to the synergistic interaction of the individual components of the extract, which increase the resulting efficiency of such complex mixtures. Hashemi and Jafarpour [61] tested the effectiveness of *Eucalyptus caesia* Benth and *Dracocephalum multicaule* complex essential oils on *E. coli*. They found that essential oils were more effective against Gram-positive than against Gram-negative bacteria. Both essential oils together showed a synergistic or additive effect.

As can be seen in Figure 1, the effect of the antimicrobial agent and boswellic acid extract on the bacteria varied; in all cases there was an additive effect of the antimicrobial agent and the natural Boswellia extract. Additive effects of erythromycin and *B. sacra* extract on gastrointestinal pathogens have also been found by Rashman [62].

Great attention is currently paid to the combinations of different natural substances and antibiotics, and the results show a significant reduction in effective antibiotic concentrations [63]. Natural substances are also effective against multi-resistant strains, such as Pseudomonas aeruginosa or MRSA [64,65,66]. Another study tested the effectiveness of salvipisone and aethiopinone against MRSA strains. These terpenes acted synergistically with oxacillin, vancomycin, and linezolid. The results of this study showed that salvipisone and aethiopinone are bactericidal or bacteriostatic against planktonic cultures of the tested bacteria [67]. Although the efficacy of the resulting combination found by us was only additive, in reality, lower antibiotic concentrations may reduce the risk of developing resistance. The method widely used by bacteria to reduce the effects of antibiotics is the use of an MDR. Some plants have MDR inhibitors and, thus, increase the activity of the antimicrobial compounds in the plants [68,69,70]. This effect can, therefore, be used when natural extracts and antibiotics are used together.

The minimum inhibitory concentration of the biofilm (MBIC50) was determined (Table 1) using the MTT test (monitoring the metabolic activity of the cells) on selected bacterial strains of *S. epidermidis*, *E. faecalis,* and *E. coli*. In ERM, the effective antibiotic concentration in Gram-positive bacteria increased by several orders of magnitude and ranged from 50–150 mg/L, in Gram-negative bacteria such as *E. coli* the effective concentration of PMB was in the range from 2–7 mg/L depending on the strain. For BOSW, the effective MBIC50 concentration ranged from 250–400 mg/L for Gram-positive bacteria and from 100–400 mg/L for Gram-negative *E. coli*. For MBIC80 values, both ERM and BOSW on Gram-positive bacteria (*S. epidermidis* and *E. faecais*) were not effective in the observed concentration range. Resistance of Enterococcus strains to common drugs is caused by naturally occurring genes for resistance to a number of antibiotic types, along with resistance acquired by horizontal genetic exchange [39]. The production of an aggregation substance, which includes a group of surface proteins encoded by a pheromone-induced conjugate plasmid, significantly contributes to the biofilm formation of these species [32]. For Gram-negative *E. coli*, PBM was effective at a concentration of 5 mg/L in the *E. coli* strain DBM 3125, for *E. coli* strains CCM 3954 and *E. coli* CCM 4517, PBM was not effective in the observed concentration range, as was BOSW. The pure form of AKBA inhibited S. epidermidis biofilm formation (MBIC50) at a concentration of 32 mg/L [71]. The high drug resistance of *E. coli* is due to the horizontal gene transfer, the ability of the microorganism to grow in the form of a biofilm, and the natural evolution of survival genes [15].

**Figure 2:**
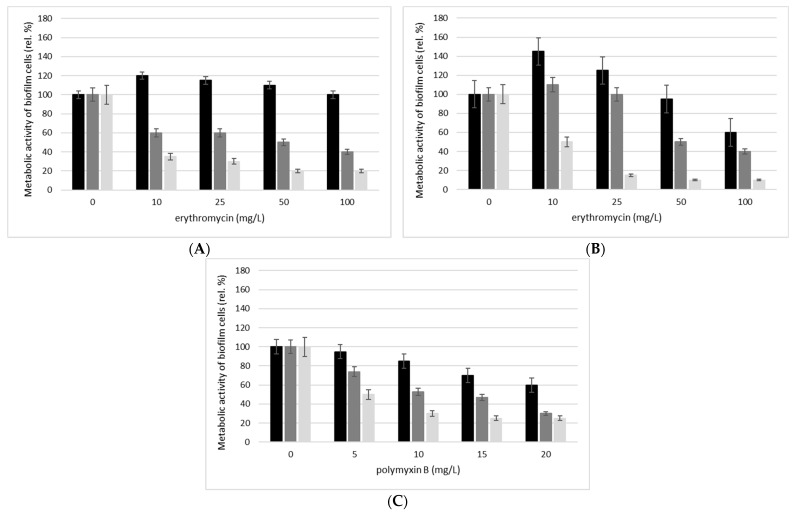
Effect of erythromycin for *S. epidermidis* and *E. faecalis* of polymixin B for *E. coli* in combination with different concentrations of boswellic acid extract (BOSW) on the metabolic activity of (**A**) *S. epidermidis* biofilm cells ATCC 14990; (**B**) *E. faecalis* biofilm cells ATCC29212; (**C**) *E. coli* biofilm cells CCM 3954. BOSW concentration: 0 mg/L (■), BOSW 1 × MIC80 (100 mg/L) (■); BOSW 1.5 × MIC80 (100 mg/L) (■); biofilm culture 24 h, 37 °C, 150 rpm, TSB medium; altering the metabolic activity of biofilm cells; expressed as a relative percentage related to the control (100%, without the influence of biological active substances).

In our work, we continued to investigate the multiple effect of the action of boswellic acids with antibiotics, in the form of inhibition of biofilm formation. The results are shown in Figure 2A–C. Based on the determined MBIC80, we tested erythromycin at concentrations of 10, 25, 50, and 100 mg/L in combination with BOSW at concentrations of 100 and 150 mg/L for the biofilm formation of Gram-positive *S. epidermidis* ATCC 14990 and *E. faecalis* ATCC29212, and for Gram-negative *E. coli* CCM 3954, a combination of BOSW, also at concentrations of 100 and 150 mg/L, and polymyxin B at concentrations of 5, 10, 15, and 20 mg/L. As can be seen, for both Gram-positive and Gram-negative representatives of the bacteria, the combinations of the two substances were effective in suppressing biofilm formation. There is currently no study testing the effect of boswellic acids in combination with antibiotics on suspension cells or on biofilm formation. However, we can compare our results with other studies such as Dimkic [72] who tested plant extracts in combination with antibiotics to inhibit biofilm formation in E. faecalis isolates. The combination of the effects of the complex plant extract and the antibiotic effectively prevented the biofilm formation of selected isolates. The addition of BOSW at a concentration of 100 mg/L reduced the metabolic activity of the monitored bacterial strains by up to 70% at the highest tested antibiotic concentration. A concentration of 150 mg/L BOSW reduced cell metabolic activity by up to 90% in combination with the highest concentration of erythromycin or polymyxin B (100 mg/L or 20 mg/L).

There are several mechanisms of antimicrobial interactions between two substances, whether it is the inhibition of protective enzymes, the sequential inhibition of a common biochemical reaction, or the use of active cell wall components that allow the antimicrobials to penetrate the cell interior more successfully [73]. In particular, members of the genus Enterococcus are characterized by high biofilm formation, especially in the presence of glucose in an environment that stimulates the synthesis of surface proteins that facilitate adhesion [74].

The significant effect of BOSW in combination with antibiotics on the inhibition of biofilm formation is likely due to the high concentration of terpene substances in the *B. serrata* extract. Terpenoids disrupt membranes, disrupt quorum sensing, inhibit protein synthesis and ATP [75]. Terpenes are phytochemicals that cause damage to the bacterial membrane, suppress some virulence factors, have significant anti-QS activity and, thus, inhibit biofilm formation. Some phytochemicals, in addition to having direct antimicrobial activity, have in vitro antibiotic resistance modifying activity when used at low MIC levels [76]. Phytocomponents of plant extracts also effectively block efflux pump inhibitors in *S. aureus* MRSA strains, thereby reducing tetracycline resistance by blocking protein efflux pumps (Tet K). The combination of tetracycline and thymol effectively reduced the number of adherent *S. aureus* cells and violated membrane integrity [77]. Di- and triterpenes have been shown to be effective antibacterial agents against both Gram-positive (*S. epidermidis*, *S. aureus*) and Gram-negative (*E. coli*) bacteria and for inhibiting biofilm formation, in combination with terpenic agents and antibiotics at subinhibitory concentrations [78]. Specifically, salvipisone and aethiopinone, two diterpenes isolated from *Salvia sclarea,* are growth inhibitors of *S. aureus*, *S. epidermidis* and *E. faecalis*. In *S. aureus*, at least 85% disrupted pre-formed biofilm [67].

Cells in the biofilm are more resistant to the effects of antimicrobials than suspension cells and the search for substances capable of dispersing cells in the biofilm is, thus, growing [79,80]. Due to the system of action of these substances, they are used in combination with an antimicrobial drug, where the dispersing agent releases the cells from the biofilm and the antibiotic kills the cells. Thus, the synergistic action of both components [81,82,83,84] is used to eradicate the biofilm. The results for biofilm eradication are interesting, as due to the high resistance of biofilm cells to antimicrobials, the MBEC80 values for BOSW reached a maximum of seven times higher values for Gram-positive cocci than the MIC80 for *S. epidermidis* and fifteen times higher for *E. faecalis* (*p* < 0.05). Additionally, for Gram-negative *E. coli*, MBEC80 values were only 3-fold higher than MIC80 (*p* < 0.05). The concentrations found by us are higher than those reported by Raja [18], who monitored the effect of AKBA on the eradication of *S. epidermidis* biofilm, where the effective concentration was 50% inhibition at 64 mg/L. However, it must be taken into account that this was the effect of pure acetyl-11-keto-β-boswellic acid. The difficulty in eradicating the bacterial biofilm is related to its arrangement. Several reasons complicating the eradication of existing biofilms are described. These include the slow and limited penetration of antimicrobials into the biofilm, a resistant phenotype with the transfer of genes and enzymes that deactivate antimicrobials, and a change in the metabolism and cellular environment of cells in the biofilm. Cells submerged deep in the biofilm are in an anoxic state, are referred to as persistent cells, and are, therefore, not sensitive to antimicrobials [85,86]. Nutrients and metabolic products are transported through the water channels of the biofilm matrix of the exopolymetric polysaccharides. Interestingly, in microbial biofilms, the pH is lower than physiological, which is outside the biofilm [87,88]. Substances capable of disintegrating the biofilm interfere with processes such as quorum sensing, which maintain the functionality of the biofilm [89].

## 3. Materials and Methods

### 3.1. Biological Active Substances

The natural boswellic acids are commercially available as Bioswellix (BOSW) (Interpharma, Prague, Czech Republic), containing acetyl-β-boswellic and acetyl-11-keto-β-boswellic acid (min. 35% *w*/*w*). The representatives of currently used antibiotics, erythromycin (ERM) and polymyxin B (PMB), were purchased from Sigma-Aldrich, Prague, Czech Republic. BOSW was dissolved in 100% dimethylsulfoxide (DMSO, Penta, Prague, Czech Republic) to a 1% maximum final concentration of DMSO in culture medium (control samples with 1% DMSO were included in all assays). Each of the antibiotics were dissolved in the appropriate growth medium.

### 3.2. Microorganisms

*Staphylococcus epidermidis* DBM 3179, *Enterococcus faecalis* DBM 3075, and *Escherichia coli* DBM 3125 were kindly provided by the Department of Biochemistry and Microbiology, UCT Prague, Czech Republic. The type strain *S. epidermidis* ATCC 14990 and *E. faecalis* ATCC 29212 were acquired from the Czech National Collection of Type Cultures (Prague, Czech Republic), strains *S. epidermidis* CCM 4418, *E. coli* CCM 3954, and *E. coli* CCM 4517 were obtained from the Czech Collection of Microorganisms (Brno, Czech Republic). All microorganisms were stored in 50% (*v*/*v*) glycerol cryopreserves at −70 °C. The microorganisms were precultured in TSB medium (Tryptone Soya Broth, Oxoid, UK) for 24 h at 37 °C and 150 rpm.

### 3.3. Cultivation of Suspension Cells

For the determination of activity against suspension cells, 30 μL of inoculum (OD600 = 0.100 ± 0.010) was added into a microtitre plate well (Honeycomb 2, Growth Curves, Piscataway, NJ, USA). After that, either fresh medium or a combination of fresh medium and antimicrobial agent solution was added to a final volume of 320 μL. Microorganisms were then cultivated in the microcultivation device, Bioscreen C (Labsystems, Vantaa, Finland), for 24 h at 37 °C. As the control, cultivation without an antimicrobial agent was used. After 24 h, the growth was evaluated as optical density at 600 nm and control cultivation assigned 100 rel. %. The lowest concentration of an agent causing 80% inhibition of suspension cells compared to the control (without the presence of antimicrobial agent), i.e., the minimum inhibitory concentration (MIC80), was then determined [90]. For each strain, the concentrations of boswellic acids were chosen from the range between 0 and 150 mg/L. The antibiotic concentrations ranged from 0 to 10 mg/L, according to the type of the antibiotic. These experiments were carried out in three independent repetitions, each of them being performed in triplicate.

Furthermore, the combined effect of boswellic acids and antibiotics was determined using the fractional inhibitory concentration index (FICi) according to Maťátková [91]. The FIC index can be calculated as follows: FICi = FICA + FICB = cA (comb.)/MIC80,A + cB/MIC80,B, where FICi is the ratio of the lowest effective concentration of the substance A and B in combination (cA (comb.) and cB (comb.)) and separate minimum inhibitory concentration of the substances (MIC80,A and MIC80,B). The index can be used for determining the relationship between the activities of the two agents. This relationship can be synergistic (FICi = 0.1–0.5), additive (FICi = 0.5–1.0), indifferent (FICi = 1.0–3.0), or antagonistic (FICi < 4.0).

### 3.4. Cultivation of Biofilm Cells

Regarding the determination of activity against adhering cells, 210 μL of inoculum (OD600 = 0.800 ± 0.020) was added to a microtitre plate well (TPP, Trasadingen, Switzerland). Growth medium and antimicrobial agent were added afterwards to make up the final volume of 280 μL. After that, the cells were cultivated for 24 h at 37 °C and 150 rpm. The activity of antimicrobial substances was explored alone and in combination. The combined effect was observed using an experiment layout according to Mishra and Wang [92]. For each strain, the particular concentrations of boswellic acids were chosen according to their respective MIC80 (100 mg/L and 150 mg/L). The antibiotic concentrations ranged from 5 to 100 mg/L, according to the type of antibiotic. These experiments were carried out in three independent repetitions, with each of them performed in quadruplicate.

### 3.5. Crystal Violet Staining

After the cultivation, total biofilm biomass was observed using crystal violet staining according to Maťátková [91]. Added to thoroughly washed biofilm was 200 μL of filtered 0.1% crystal violet solution (Carl Roth, Germany), which was incubated for 20 min at room temperature. Subsequently, the unbound stain was washed out carefully with saline solution and the dye bound to the biofilm was extracted using 200 μL of 96% ethanol (10 min at room temperature). Then, 100 μL was taken from each well and absorbance was measured at 580 nm. The results are shown as the relative percentage (with the control experiment representing 100%). The experiments were carried out in three independent repetitions, with each of them performed in quadruplicate.

### 3.6. Metabolic Activity of Cells in Biofilm

For the metabolic activity determination, the biofilm was evaluated using viability assay mediated with MTT according to Riss (2013) [93]. First, the biofilm was washed with sterile phosphate buffer saline (PBS, pH 7.4). After that, 50 μL of MTT (1 g/L of 3-(4,5-dimethylthiazol-2-yl)-2,5-diphenyltetrazolium bromide; Across Organics, Geel, Belgium) dissolved in PBS, filtered through a 0.22 μm filter (EDM Millipore, Burlington, MA, USA), and 60 μL of D-glucose (57.4 g/L) dissolved in PBS was added into each well. The cells were incubated for 1 h at 37 °C and 150 rpm. Crystals of formazan created by enzymes provided by the living biofilm cells were dissolved using 100 μL of wash-out solution. The solution contained 6 parts (*v*/*v*) of PBS containing 2% acetic acid (Penta, Prague, Czech Republic) and 4 parts (*v*/*v*) dimethylformamide (Carl Roth, Germany), at the end 16% (*w*/*v*) of sodium dodecylsulphate (Carl Roth, Germany) was edded. To dissolve the crystals, the plate was kept at 230 rpm for 30 min at room temperature. 100 μL was taken from each well and absorbance was measured at 570 nm.

The results of the viability assay were used for the determination of the minimum biofilm inhibitory concentration (MBIC50 and MBIC80), which is defined as the lowest concentration lowering the metabolic activity of cells in biofilm by 50 or 80%, respectively, in comparison to the control after 24 h of cultivation. Similarly, this was also assessed in the context of a combination. A ratio of boswellic acids and antibiotic, which led to achieving 80% inhibition was sought. The experiments were carried out in three independent repetitions, with each of them performed in quadruplicate.

### 3.7. Minimum Biofilm Eradication Concentrations

Biofilm eradication activity was assessed using pre-cultivated mature biofilm, which was cultivated in the absence of an antimicrobial substance (OD600 = 0.600 ± 0.020) for 24 h at 37 °C and 150 rpm. Then, the biofilm was washed three times with physiological saline solution and cultivated for another 24 h in the presence of an antimicrobial agent. The concentrations of an antimicrobial substance inhibiting the biofilm cells by 50 or 80% were interpreted as minimum biofilm eradication concentration (MBEC50 and MBEC80, respectively). The experiments were performed in 8 parallels.

### 3.8. Statistical Analysis

The distant results were identified and omitted according to Dixon’s Q Test. The arithmetic mean and standard deviation were calculated from colorimetric data. The significance of results was evaluated by one-way analysis of variance (ANOVA) with a significance level of *p* < 0.05.

## 4. Conclusions

Increasing bacterial resistance to known antibiotics has been a growing problem in recent times. Bacteria that are capable of forming a biofilm are even more resistant to inactivation. Due to this fact, it is necessary to search for new substances that would support the elimination of these biofilm-forming microorganisms. Plant extracts and their essential oils are often associated with a positive effect on the inactivation of biofilm-forming bacteria. Extracts of *Boswellia* sp. plants containing highly biologically active boswellic acids could be effective this way, as confirmed by this study. Their effect of acting synergistically with commonly used antibiotics was demonstrated in all strains of *Staphylococcus epidermidis*, *Enterococcus faecalis*, and *Escherichia coli* tested. For this reason, it would be interesting to continue to research these acids and to expand their use as additives to commercially available antimicrobials.

## Figures and Tables

**Table 1:** Effect of antibiotics (erythromycin, polymyxin B) and boswellic acids extraction microbial suspension growth, biofilm formation, and biofilm eradication.

Antimicrobial and Antibiofilm Activity of ERM, PMB, and BOSW							
		MIC50	MIC80	MBIC 50	MBIC 80	MBEC 50	MBEC 80
		(mg/L)	(mg/L)	(mg/L)	(mg/L)	(mg/L)	(mg/L)
*S. epidermidis* DBM 3179	ERM	0.5	1	150 ^a^	400 ^a^	30	70
	BOSW	70	100	300	500 ^a^	150	500
*S. epidermidis* CCM 4418	ERM	0.3	0.5	100	400 ^a^	150 ^a^	150 ^a^
	BOSW	50	80	250	500	100	400
*S. epidermidis* ATCC 14990	ERM	0.45	0.75	100	400 ^a^	150 ^a^	150 ^a^
	BOSW	50	70	300	500 ^a^	150	450
*E. faecalis* DBM 3075	ERM	0.5	1	50	400 ^a^	15	150 ^a^
	BOSW	10	25	400	500 ^a^	100	300
*E. faecalis* ATCC 29212	ERM	0.5	1	60	400 ^a^	60	150 ^a^
	BOSW	10	20	300	500	110	350
*E. coli* DBM 3125	PBM	0.5	0.7	2	5	1	3
	BOSW	100	150	100	500 ^a^	100	300
*E. coli* CCM 3954	PMB	0.4	0.7	5	20 ^a^	3	20 ^a^
	BOSW	100	130	400	500	300	500
*E. coli* CCM 4517	PMB	0.4	0.8	7	20 ^a^	3	20 ^a^
	BOSW	80	120	300	500	250	500

ERM erythromycin, PMB polymyxin B, BOSW extract from *B. serrata*, MIC50 minimum inhibitory concentrations (50%), MIC80 minimum inhibitory concentrations (80%), MBIC50 minimum biofilm inhibitory concentrations (50%), MBIC80 minimum biofilm inhibitory concentrations (80%), MBEC50 minimum biofilm eradication concentrations (50%), MBEC80 minimum biofilm eradication concentrations (80%), ^a^ Not determined at the highest tested concentration. All results were significant according to ANOVA (*p* < 0.05).

## Data Availability

Not applicable.

## Sample Availability

Samples of the compounds are available from the authors.
